# Triazine-ring protonation enables synergistic enhancement of proton conduction and membrane stability

**DOI:** 10.1039/d5sc05445a

**Published:** 2025-10-31

**Authors:** Yunfa Dong, Haodong Xie, Yupei Han, Quan Li, Jiecai Han, Weidong He

**Affiliations:** a National Key Laboratory of Science and Technology on Advanced Composites in Special Environments, and Center for Composite Materials and Structures, Harbin Institute of Technology Harbin 150080 China weidong.he@hit.edu.cn; b Department of Chemistry, University College London London WC1H 0AJ UK; c Chongqing Academy of Science and Technology Chongqing 401100 China; d Chongqing Research Institute, Harbin Institute of Technology Chongqing 401151 China

## Abstract

The power conversion efficiency of proton exchange membrane fuel cells (PEMFCs) is directly determined by proton-conduction-attributed current across conventional perfluorosulfonic acid (PFSA) membranes. Incorporation of fillers is frequently proposed to increase the in-membrane active sites and proton conduction, but the enhancement typically sacrifices membrane stability due to the phase separation and structural defects introduced by the fillers. Herein, we integrate a high polarity supramolecular complex, specifically melamine trithiocyanuric acid (MT), into PFSA to create a homogeneous composite proton exchange membrane (PEM) using molecular-level hybridization. In the composite PEM, the protonated triazine ring (PTR) of MT forms heterogeneous distribution regions featuring SO_3_^−^–H_3_O^+^–PTR interfaces with hydrated PFSA. This phenomenon arises from the alkaline property of pyridine-like nitrogen, whose lone-pair electrons do not participate in the conjugated system. These regions not only provide a facile proton transfer pathway with a high mean square displacement (MSD) of 1.63 × 10^−8^ cm^2^ s^−1^, but also enhance proton conduction *via* the Grotthuss mechanism through moderately strengthened, dynamically stable multiple hydrogen-bond interactions. This composite PEM demonstrates an excellent proton conductivity of 0.249 S cm^−1^ at 90 °C, and achieves a power density of 1416 mW cm^−2^ at 70 °C in a hydrogen fuel cell. The 72-hour Fenton degradation test exhibits a mass loss of only 13.8 wt%.

## Abstract 2

The protonated triazine ring (PTR) in melamine trithiocyanuric acid forms heterogeneous domains, creating SO_3_^−^–H_3_O^+^–PTR interfaces with hydrated perfluorosulfonic acid that synergistically enhance proton conduction and membrane stability.

## Graphical abstract

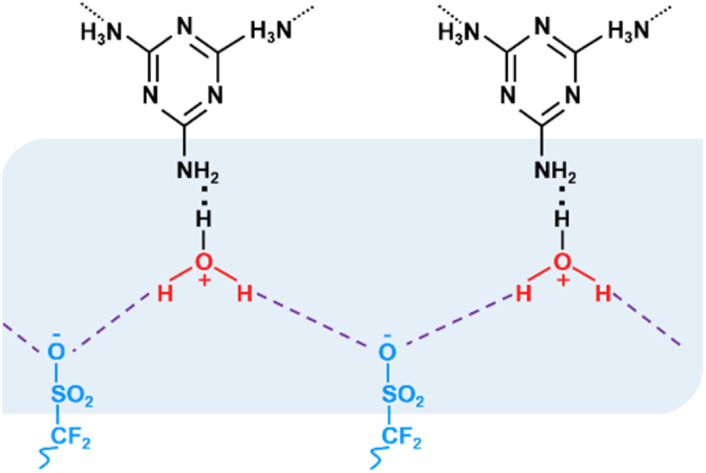

## Introduction

Amidst the global fossil energy crisis and concerns regarding the greenhouse effect, hydrogen energy applications hold significant promise due to their high energy conversion efficiency and minimal environmental impact.^1,2^ Proton exchange membrane fuel cells (PEMFCs) represent a commercially successful power generation technology in hydrogen energy applications. The proton exchange membrane (PEM) serves as the locus for hydrogen fuel cell power generation reactions, with key parameters such as proton conductivity critically influencing power density and fuel cell lifespan.^3^ With the increasing diversification of fuel cell applications, the demand for high power density (≥2000 W kg^−1^) underscores the urgent need for enhancing the proton conductivity (≥0.2 S cm^−1^) of PEMs.^4^

Perfluorosulfonic acid (PFSA) membranes stand as the predominant substrate for PEMs, with brands like DuPont, Gore-Select, and Asahi Glass being globally commercialized due to their robust stability and exceptional mechanical properties. Nonetheless, significant shortcomings persist, including low proton conductivity under high temperature/low humidity conditions, elevated hydrogen permeability and swelling rates, and diminished thermal stability and water uptake rates under long-term service.^5,6^ Employing an inorganic–organic composite strategy to modify PFSA-based PEMs has proven effective, involving materials like carbon quantum dots,^2^ bismuth oxide clusters,^7^ and polyoxometalate,^8^ among others, to functionalize PEMs. However, the inherently weak physical interaction between fillers and the polymer matrix often leads to phase separation issues such as random distribution, agglomeration, and delocalization of fillers during reactions, hampering the attainment of homogeneous membranes.^9^ Additionally, conventional polymer compounding strategies frequently diminish the proton conductivity of composite membranes, with poor compatibility between polymers exacerbating challenges such as phase separation during prolonged usage.^10^ Consequently, the designation of fillers with proton conduction activity while ensuring molecular-level hybridization and high membrane stability with PFSA-based PEMs poses a formidable challenge.

Addressing the phase separation and proton inertness of rigid fillers, this work proposes a molecular engineering design strategy involving soft material–supramolecular complex composite PFSA. Supramolecular complexes offer advantages such as flexible molecular structure design, straightforward synthesis processes, facile protonation of surface functional groups, and ample intramolecular free volume.^11^ Utilizing intermolecular hydrogen bonding as the driving force, the three –NH_2_ groups on the triazine ring of melamine and the three –SH groups on the triazine ring of trithiocyanuric acid undergo a complete reaction to form an ammonium salt, thereby protonating the triazine ring and prompting melamine trithiocyanuric acid (MT) self-assembly into supramolecular complexes. In the composite PEM, the protonated triazine ring (PTR) of MT forms heterogeneous distribution regions featuring SO_3_^−^–H_3_O^+^–PTR interfaces with hydrated PFSA. This phenomenon arises from the alkaline property of pyridine-like nitrogen, whose lone pair electrons do not participate in the conjugated system. This study harnesses the PTR to introduce additional proton bridging to the composite PEM, establishing multiple proton transfer channels, and enhancing proton conductivity. Furthermore, the hydrogen bonding interaction of the supramolecular complex acts as a ‘moisturizing factor’ for the composite membrane, augmenting water absorption and expanding the hydration area, thereby further improving proton conductivity. The coupling interaction and molecular-level hybridization between PFSA and supramolecular complexes also enhance the tensile strength and membrane stability of the composite PEM while reducing hydrogen permeability. The composite PEM exhibits a remarkable proton conductivity of up to 0.249 S cm^−1^ at 90 °C and a power density of 1416 mW cm^−2^ at 70 °C in a hydrogen fuel cell, and the 72-hour Fenton degradation test exhibits a mass loss of only 13.8 wt%, presenting a novel approach for constructing a composite PEM with multi-stage proton transfer channels.

## Results and discussion

**Fig. 1:**
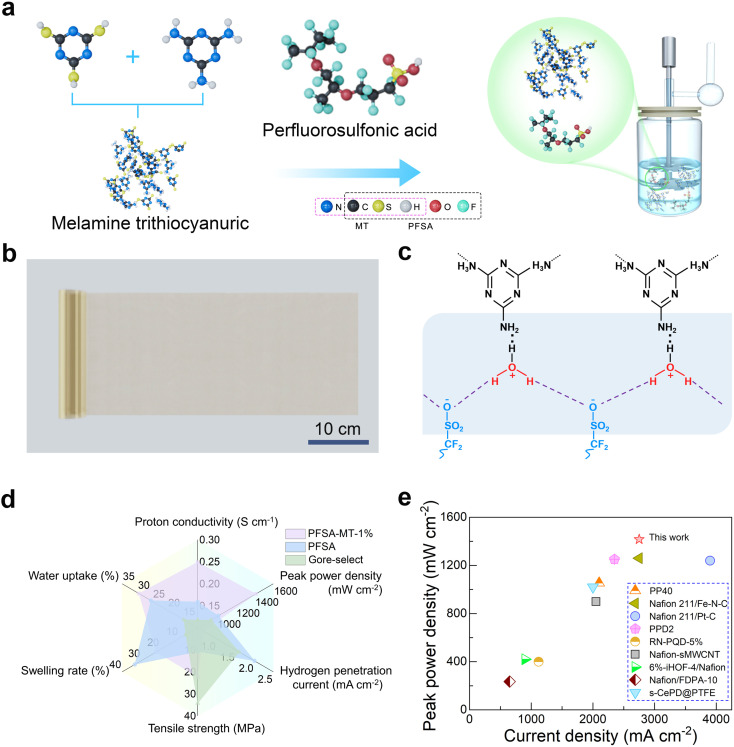
PEM preparation process, proton conduction mechanism, and performance comparison. (a) Flowchart of the composite proton exchange membrane slurry fabrication process. (b) Digital photograph of large-scale composite PEMs. (c) Schematic diagram of the formation of additional hydrogen bonds between PTR and hydrated PFSA, and the proton conduction mechanism. (d) A parameter comparison radar chart. (e) Comparative data of different PEMs in related literature studies.

As shown in Fig. 1a and S1, MT is synthesized through a one-step, H_2_O-assisted reaction between melamine and trithiocyanuric acid, facilitated by hydrogen bonding interactions.^12^ This process facilitates the modulation of the MT molecular structure by regulating the molar ratio of reactants. Fig. 1b portrays a large-format PFSA-MT-1% composite membrane, evidencing a viable technical route for mass production.

The proton conduction mechanism of the composite PEM is depicted in Fig. 1c and S2. First, the PFSA membrane features a singular conventional proton conduction path. Upon incorporating MT, the protonated triazine ring (NH_3_^+^) within MT interacts with the sulfonate group SO_3_H^−^ in hydrated PFSA through H_2_O, forming additional hydrogen bonding interactions. Consequently, the composite membrane develops multi-stage proton transfer channels, enhancing proton conduction efficiency. The integration of MT results in a denser composite membrane structure, effectively impeding hydrogen permeation. Moreover, the hydrogen bonding interaction between MT and PFSA acts as a “moisturizing factor” for the composite membrane, augmenting its water absorption capacity and expanding the hydration area, thus further enhancing proton conductivity. Fig. 1d presents a parameter comparison among PFSA-MT-1%, PFSA, and Gore-Select membranes. While PFSA-MT-1% exhibits superior overall electrochemical performance, its swelling rate and tensile strength lag behind those of commercial membranes, indicating areas for improvement. Comparative data in Fig. 1e highlight PFSA-MT-1%'s advantages in current density and power density over similar previously reported PEMs.^2,10,13–21^

**Fig. 2:**
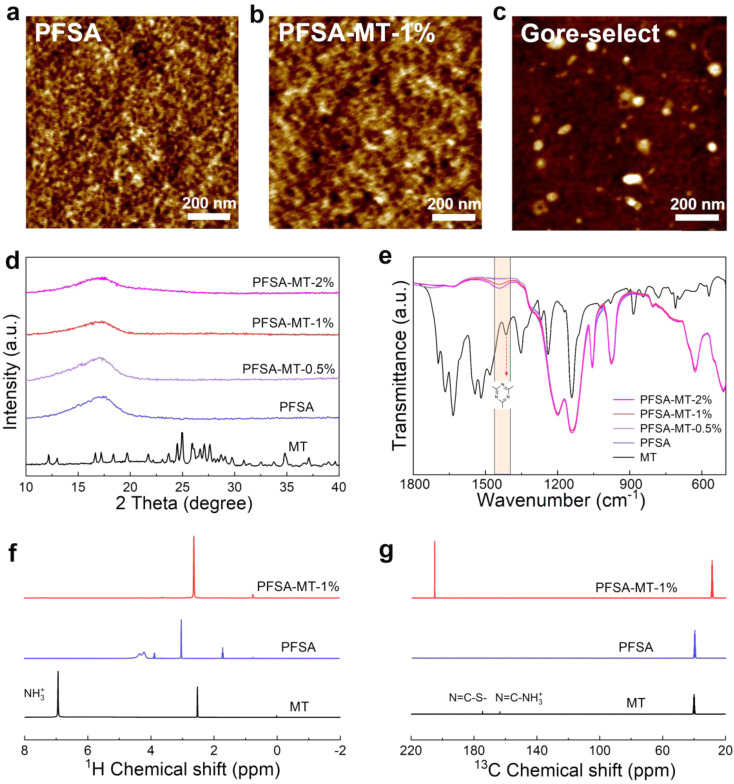
The physicochemical properties of PEMs. AFM phase images of different proton exchange membranes: (a) PFSA, (b) PFSA-MT-1%, and (c) Gore-Select. (d) XRD spectra of MT and different membranes. (e) FT-IR spectra of MT and different membranes. (f) ^1^H, and (g) ^13^C liquid-state NMR spectra of MT, PFSA and PFSA-MT-1%.

Atomic force microscopy (AFM) was used to probe the nano-phase separation structure of composite PEMs. As shown in Fig. 2a, under hydrated conditions, PFSA exhibits a distinct surface morphology “fingerprint image”, where the dark and bright regions correspond to the hydrophilic ionic nano-phase and the hydrophobic backbone nano-phase, respectively.^22^ This unique hierarchical structure is formed through entropy-driven self-assembly of amphiphilic perfluorosulfonic acid chains and the pre-arrangement of chains in the casting solution. As shown in Fig. 2b, after adding MT, the “fingerprint-like” structure of PFSA was retained with slight weakening, and clusters of hydrophilic dark regions showed significant enlargement, which is beneficial for enhancing the proton conductivity of composite PEMs. For PFSA-MT-1%, due to the high miscibility between MT and PFSA, it is difficult to distinguish the continuous ionic nano-phase cut into isolated large-scale domains by MT, demonstrating the integrated compatibility advantage of alloy membranes. The nanoscale size and polarity matching between MT and PFSA nano-phase maintains the required phase separation. Additionally, as shown in Fig. 2a–c, and S3, PFSA-MT-1% exhibits lower surface roughness compared to the commercial Gore-Select membrane, which indicates a high degree of molecular-level hybridization in the alloy membrane. This characteristic enhances the mechanical strength of the composite proton exchange membrane and facilitates subsequent membrane–electrode assembly fabrication.

Besides the hydrophilic–hydrophobic properties and surface roughness observed by AFM mentioned above, membrane surface properties also play an important role in proton conduction. Contact angle images of different PEMs are shown in Fig. S4. The test results demonstrate that the addition of MT induces a slight increase in the contact angle of the PFSA membrane. However, this does not compromise the overall hydrophilic characteristics of the proton exchange membrane. The incorporation of MT promotes the formation of a built-in 3D reinforced hydrogen bond network within PFSA, which enhances the water uptake of the composite proton exchange membrane. Consequently, this improvement elevates the proton conductivity of the membrane and ultimately boosts the power density of hydrogen fuel cells.

MT exhibits a certain solubility in NMP and high polarity, enabling its complete miscibility with PFSA in NMP and achieving molecular-level hybridization during subsequent homogeneous membrane casting. X-ray diffraction (XRD) spectra data reveal that the addition of MT does not significantly alter the original PFSA structure, with no characteristic MT crystal peaks detected in the composite membrane (Fig. 2d). This absence of distinct peaks may be attributed to MT's molecular-level hybridization with PFSA within the composite membrane, leading to its amorphous characteristics. Furthermore, as the MT content increases, the crystallinity of the composite membrane gradually decreases. This indicates that more hydrogen bonding interactions form at the SO_3_^−^–H_3_O^+^–PTR interfaces, altering the polymer packing arrangement and thereby reducing its crystallinity.^22^ The small-angle X-ray scattering (SAXS) patterns of the three membrane materials are shown in Fig. S5. The scattering peak near 0.1 nm^−1^ typically corresponds to the periodic structure of sulfonic acid group-enriched regions in the PEM. The shift of this peak toward higher *q*-values indicates a reduction in the average distance between hydrophilic ionic clusters within the composite PEM, revealing the possibility of shortened proton conduction pathways. This results in shorter, more densely packed, and continuous proton transport channels, potentially enhancing proton conductivity through the Grotthuss mechanism. Meanwhile, the increased *q* value is also consistent with the data showing that the MT-restricted composite membrane swells and its swelling rate decreases.

Fig. 2e illustrates the application of Fourier transform infrared spectroscopy (FT-IR) to elucidate functional groups and molecular interactions. In the MT spectrum, the sharp absorption peak at 1544 cm^−1^ corresponds to the characteristic NH_3_^+^ peak, indicating the complete reaction of melamine and trithiocyanuric acid and the formation of an ammonium salt. Peaks at 1636 cm^−1^, 1543 cm^−1^, and 1518 cm^−1^ are attributed to the skeleton vibration characteristic peaks of the C=N bond of the triazine ring.^23^ In PFSA, peaks at 1200 cm^−1^ and 1141 cm^−1^ correspond to the asymmetric and symmetric stretching of the C–F bond in polytetrafluoroethylene (PTFE), respectively, while the symmetric stretching of S–O and C–O–C in the PFSA side chain occur at 1055 cm^−1^ and 975 cm^−1^, respectively.^2^ Notably, a more pronounced skeletal vibration peak of the triazine ring (1414 cm^−1^) is observed in PFSA-MT-1% and PFSA-MT-2%, accompanied by a blue shift as MT content increased in the composite membrane's peak position, indicating the formation of strong hydrogen bonding interactions by the triazine ring within the composite membranes through Lewis acid–base interaction.

Nuclear magnetic resonance (NMR) of three materials is presented in Fig. 2f and g. As shown in Fig. 2f, the absorption peak at 6.94 ppm of MT is the proton characteristic peak of NH_3_^+^, indicating the formation of a protonated triazine ring. The absorption peaks at 174.79 ppm and 163.55 ppm of MT correspond to N=C–S^−^ and N=C–NH_3_^+^ related to the triazine ring, respectively (Fig. 2g).^24^ The deviation of the PFSA-MT-1% absorption peak from a simple MT/PFSA superposition points to potential strong hydrogen bonding and other interactions. Furthermore, this molecular hybridization effectively impedes hydrogen penetration while bolstering tensile strength (22.3 MPa, Fig. S6).

**Fig. 3:**
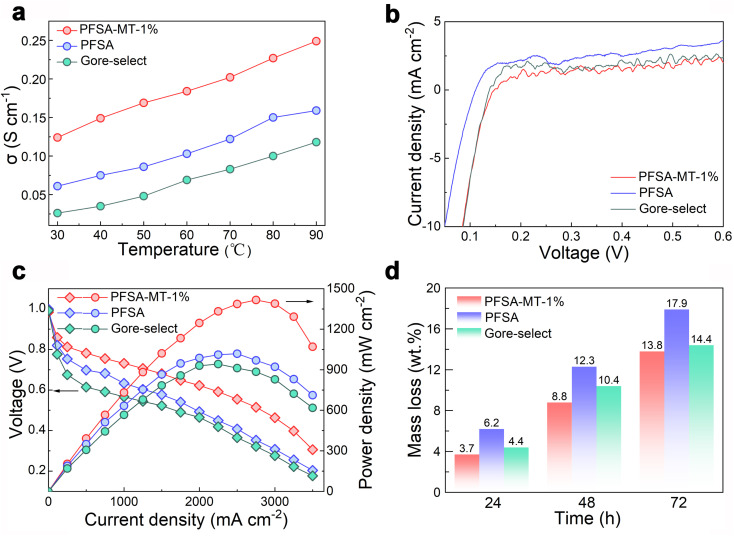
The electrochemical performance of PEMs. (a) Proton conductivity of different membranes at different temperatures. (b) Hydrogen permeation current density of different membranes. (c) Polarization, and power density curves of the composite membrane; MEAs' test environment is 70 °C and 100% RH. (d) *Ex situ* degradation experiments results at different testing times.

Factors influencing proton conductivity encompass the proton transfer channels within the composite PEM and the water absorption rate. Fig. 3a and S7 compares the proton conductivity of PEMs across different temperatures. The data reveal that PFSA-MT-1% exhibits the highest proton conductivity within the 30–90 °C range, notably reaching 0.249 S cm^−1^ at 90 °C, attributed to the protonated triazine ring from MT. Strong hydrogen bonding interactions between the PTR and hydrated PFSA's SO_3_H significantly widen the proton transfer channels. Moreover, as shown in Fig. S8, we conducted time-dependent conductivity measurements at 90 °C. The test results show that even after 96 hours of continuous exposure to a high temperature of 90 °C under hydrated conditions, PFSA-MT-1% still maintained a high proton conductivity of 0.248 S cm^−1^, corresponding to a retention rate of 99.6%, indicating its favorable durability and practicality. In addition, as shown in Fig. S9, the thermogravimetric analysis (TGA) curve demonstrates that PFSA-MT-1% exhibits excellent stability below 300 °C, while the differential scanning calorimetry (DSC) curve indicates that its peak mass loss temperature occurs at 487 °C, both of which are significantly higher than its maximum practical operating temperature of 90 °C. Furthermore, the aforementioned time-dependent conductivity measurements at 90 °C also confirm its outstanding long-term proton conduction and thermal stability. Furthermore, an optimal composite PEM necessitates a dense structure to minimize hydrogen permeation, ensuring hydrogen fuel cell safety. As depicted in Fig. 3b, PFSA-MT-1% exhibits a hydrogen permeation current of only 0.65 mA cm^−2^, presenting advantages over PFSA (2 mA cm^−2^) and Gore-Select (1.6 mA cm^−2^). This advantage stems from MT-PFSA molecular-level hybridization, enhancing composite membrane density and effectively restraining hydrogen penetration.

Fig. 3c delineates the polarization and power density curves of the composite membrane. PFSA-MT-1% exhibits minimal ohmic polarization and the slowest voltage decay rate. With a high power density of 1416 mW cm^−2^ at 70 °C in a hydrogen fuel cell, surpassing PFSA (1019 mW cm^−2^) and Gore-Select (942 mW cm^−2^), PFSA-MT-1% offers evident practical advantages. Transition metal ions such as Fe^2+^ can catalyze the decomposition of H_2_O_2_ into oxygen radicals through the Fenton reaction. The chemical degradation of PEMs can be significantly accelerated by immersing the composite membrane in a H_2_O_2_ solution containing Fe^2+^ for *ex situ* degradation experiments.^25,26^ As shown in Fig. 3d, the addition of MT significantly reduces the degradation rate of PFSA even in the degradation experiment with 72 h of soaking. The mass loss of PFSA-MT-1% is 13.8 wt%, which is lower than that of PFSA (17.9 wt%) and Gore-Select (14.4 wt%). This may be due to the addition of MT to the formation of intermolecular hydrogen bonding and the denser membrane structure, which improves the antioxidant capability and effectively delays the degradation rate of the composite membrane. Furthermore, we conducted inductively coupled plasma mass spectrometry (ICP-MS) analysis of the fluorine element content in water where the composite membrane had been continuously immersed at 90 °C for 96 hours. The test results indicated no detectable fluorine element in the water, demonstrating that no PFSA leaching occurred from the composite membrane. This finding is consistent with its excellent Fenton reaction test performance, which is attributed to the polarity matching between PFSA and MT as well as their molecular-level hybridization, ensuring the integrity and durability of the composite membrane. Moreover, comparative data in Fig. 1e highlight PFSA-MT-1%'s advantages in current density and power density over similar previously reported PEMs.^2,10,13–21^

**Fig. 4:**
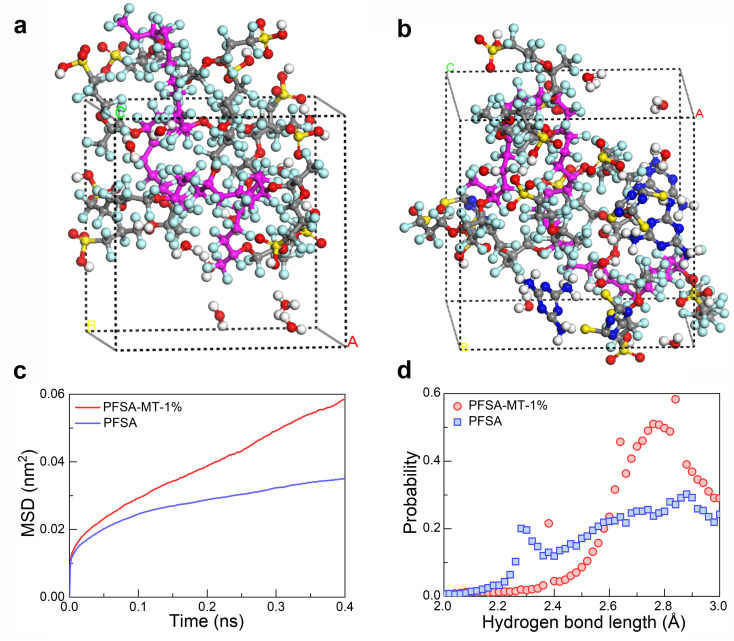
Molecular dynamics simulation. Simulation snapshot of molecular dynamics: (a) PFSA, and (b) PFSA-MT-1%. (c) Proton MSD, and (d) probability distributions of hydrogen bond lengths in PFSA and PFSA-MT-1%.

Fig. 4a and b display molecular dynamics simulation snapshots, presenting the simulated mean square displacement (MSD) of ions and the probability distribution of hydrogen bond lengths for both PFSA and PFSA-MT-1%.^27,28^ The simulation results in Fig. 4c indicate that PFSA-MT-1% exhibits a higher ionic mean squared displacement (1.63 × 10^−8^*vs.* 7.07 × 10^−9^ cm^2^ s^−1^) compared to PFSA, which is consistent with the previously mentioned high proton conductivity and high power density data. Furthermore, Fig. 4d demonstrates PTR-induced reinforcement of hydrogen bonding in the composite PEM. The typical hydrogen bond length distribution of PFSA-MT-1% exhibits a peaks at 2.75 Å (*vs.* 2.9 Å for pristine PFSA), confirming that PTR optimizes the percolation of 3D H-bond networks and increases bond density. These structural enhancements facilitate proton hopping *via* the Grotthuss mechanism.

## Conclusion

In summary, this work demonstrates a facile and large-scale production method for preparing PTR composite PFSA PEMs with multi-level proton transfer channels. Within the composite PEM, the PTR of MT establishes heterogeneously distributed domains characterized by SO_3_^−^–H_3_O^+^–PTR interfaces with hydrated PFSA. This phenomenon stems from the alkaline property of pyridine-like nitrogen, where its lone pair electrons do not engage in the conjugated system. Molecular hybridization increases the relative density of the membrane structure, thereby reducing the hydrogen permeation of the composite PEM and improving the resistance of the composite membrane to free radical oxidation. This composite PEM demonstrates an excellent proton conductivity of 0.249 S cm^−1^ at 90 °C, and achieves a power density of 1416 mW cm^−2^ at 70 °C in a hydrogen fuel cell. The 72-hour Fenton degradation test exhibits a mass loss of only 13.8 wt%. It provides a new practical strategy for the manufacturing of hydrogen fuel cells with low-cost, high-power density and long-lifespan.

## Author contributions

Yunfa Dong: conceptualization, methodology, investigation, data curation, visualization, writing – original draft, writing – review & editing. Haodong Xie: software, writing – review & editing. Yupei Han: writing – review & editing. Quan Li: writing – review & editing. Jiecai Han: writing – review & editing. Weidong He: conceptualization, methodology, supervision, funding acquisition, writing – review & editing.

## Conflicts of interest

There are no conflicts to declare.

## Supplementary Material

SC-016-D5SC05445A-s001

## Data Availability

The data that support the findings of this study are available from the corresponding author upon reasonable request. Supplementary information is available. See DOI: https://doi.org/10.1039/d5sc05445a.

## References

[cit1] Dong Y., Zhong S., He Y., Liu Z., Zhou S., Li Q., Pang Y., Xie H., Ji Y., Liu Y., Han J., He W. (2024). Modification strategies for non-aqueous, highly proton-conductive benzimidazole-based high-temperature proton exchange membranes. Chin. Chem. Lett..

[cit2] Wu W., Li Y., Liu J., Wang J., He Y., Davey K., Qiao S.-Z. (2018). Molecular-level hybridization of Nafion with quantum dots for highly enhanced proton conduction. Adv. Mater..

[cit3] Jiao K., Xuan J., Du Q., Bao Z., Xie B., Wang B., Zhao Y., Fan L., Wang H., Hou Z., Huo S., Brandon N. P., Yin Y., Guiver M. D. (2021). Designing the next generation of proton-exchange membrane fuel cells. Nature.

[cit4] Guo Y., Jiang Z., Ying W., Chen L., Liu Y., Wang X., Jiang Z.-J., Chen B., Peng X. (2018). A DNA-threaded ZIF-8 membrane with high proton conductivity and low methanol permeability. Adv. Mater..

[cit5] Feng C., Dong Y., Zhong S., Chen D., Zeng G., He W. (2022). Optimizing the molecular weight of poly(vinylidene fluoride) for competitive perfluorosulfonic acid membranes. Phys. Status Solidi RRL.

[cit6] Guan P., Zou Y., Zhang M., Zhong W., Xu J., Lei J., Ding H., Feng W., Liu F., Zhang Y. (2023). High-temperature low-humidity proton exchange membrane with “stream-reservoir” ionic channels for high-power-density fuel cells. Sci. Adv..

[cit7] Liu B., Hu B., Du J., Cheng D., Zang H.-Y., Ge X., Tan H., Wang Y., Duan X., Jin Z., Zhang W., Li Y., Su Z. (2021). Precise molecular-level modification of Nafion with bismuth oxide clusters for high-performance proton-exchange membranes. Angew. Chem., Int. Ed..

[cit8] Kim Y., Ketpang K., Jaritphun S., Park J. S., Shanmugam S. (2015). A polyoxometalate coupled graphene oxide–Nafion composite membrane for fuel cells operating at low relative humidity. J. Mater. Chem. A.

[cit9] Vinothkannan M., Hariprasad R., Ramakrishnan S., Kim A. R., Yoo D. J. (2019). Potential bifunctional filler (CeO_2_–ACNTs) for Nafion matrix toward extended electrochemical power density and durability in proton-exchange membrane fuel cells operating at reduced relative humidity. ACS Sustain. Chem. Eng..

[cit10] Ru C., Gu Y., Duan Y., Zhao C., Na H. (2019). Enhancement in proton conductivity and methanol resistance of Nafion membrane induced by blending sulfonated poly(arylene ether ketones) for direct methanol fuel cells. J. Membr. Sci..

[cit11] Wang Z.-Q., Wang X., Yang Y.-W. (2024). Pillararene-based supramolecular polymers for adsorption and separation. Adv. Mater..

[cit12] Wu M., Liu Y., Zhu Y., Lin J., Liu J., Hu H., Wang Y., Zhao Q., Lv R., Qiu J. (2017). Supramolecular polymerization-assisted synthesis of nitrogen and sulfur dual-doped porous graphene networks from petroleum coke as efficient metal-free electrocatalysts for the oxygen reduction reaction. J. Mater. Chem. A.

[cit13] Xu X.-Q., Cao L.-H., Yang Y., Zhao F., Bai X.-T., Zang S.-Q. (2021). Hybrid Nafion membranes of ionic hydrogen-bonded organic framework materials for proton conduction and PEMFC applications. ACS Appl. Mater. Interfaces.

[cit14] Yoon K. R., Lee K. A., Jo S., Yook S. H., Lee K. Y., Kim I.-D., Kim J. Y. (2019). Mussel-inspired polydopamine-treated reinforced composite membranes with self-supported CeO_x_ radical scavengers for highly stable PEM fuel cells. Adv. Funct. Mater..

[cit15] Liu L., Huang A., Yang J., Chen J., Fu K., Sun W., Deng J., Yin J.-F., Yin P. (2024). Supramolecular complexation of metal oxide cluster and non-fluorinated polymer for large-scale fabrication of proton exchange membranes for high-power-density fuel cells. Angew. Chem., Int. Ed..

[cit16] Liu S., Meyer Q., Jia C., Wang S., Rong C., Nie Y., Zhao C. (2023). Operando deconvolution of the degradation mechanisms of iron–nitrogen–carbon catalysts in proton exchange membrane fuel cells. Energy Environ. Sci..

[cit17] Macauley N., Lousenberg R. D., Spinetta M., Zhong S., Yang F., Judge W., Nikitin V., Perego A., Qi Y., Pedram S., Jankovic J., Zenyuk I. V., Xu H. (2022). Highly durable fluorinated high oxygen permeability ionomers for proton exchange membrane fuel cells. Adv. Energy Mater..

[cit18] Liu Q., Liu X., Zheng L., Shui J. (2018). The solid-phase synthesis of an Fe-N-C electrocatalyst for high-power proton-exchange membrane fuel cells. Angew. Chem., Int. Ed..

[cit19] Li Y., Wu H., Yin Y., Cao L., He X., Shi B., Li J., Xu M., Jiang Z. (2018). Fabrication of Nafion/zwitterion-functionalized covalent organic framework composite membranes with improved proton conductivity. J. Membr. Sci..

[cit20] Steffy N. J., Parthiban V., Sahu A. K. (2018). Uncovering Nafion-multiwalled carbon nanotube hybrid membrane for prospective polymer electrolyte membrane fuel cell under low humidity. J. Membr. Sci..

[cit21] Zhang H., Hu Q., Zheng X., Yin Y., Wu H., Jiang Z. (2019). Incorporating phosphoric acid-functionalized polydopamine into Nafion polymer by *in situ* sol-gel method for enhanced proton conductivity. J. Membr. Sci..

[cit22] Smith K., Foglia F., Clancy A. J., Brett D. J. L., Miller T. S. (2023). Nafion matrix and ionic domain tuning for high-performance composite proton exchange membranes. Adv. Funct. Mater..

[cit23] Zhang Q., Zhou J., Zhang H., Qi C., Zhou Q., Guo R., Yang H., Xing T., Wang M., Wu M., Wu W. (2024). Terphenyl functionalized covalent triazine polymer as metal-free photocatalyst for superior hydrogen peroxide production. Adv. Funct. Mater..

[cit24] Wang N., Peng Z. (2016). Preparation of melamine trimeric thiocyanate. Chin. J. Synth. Chem..

[cit25] Pozio A., Silva R. F., De Francesco M., Giorgi L. (2003). Nafion degradation in PEFCs from end plate iron contamination. Electrochim. Acta.

[cit26] Inaba M., Kinumoto T., Kiriake M., Umebayashi R., Tasaka A., Ogumi Z. (2006). Gas crossover and membrane degradation in polymer electrolyte fuel cells. Electrochim. Acta.

[cit27] He H., Zhu Y., Li T., Song S., Zhai L., Li X., Wu L., Li H. (2022). Supramolecular anchoring of polyoxometalate amphiphiles into Nafion nanophases for enhanced proton conduction. ACS Nano.

[cit28] He H., Song S., Zhai L., Li Z., Wang S., Zuo P., Zhu Y., Li H. (2024). Supramolecular modifying Nafion with fluoroalkyl-functionalized polyoxometalate nanoclusters for high-selective proton conduction. Angew. Chem., Int. Ed..

