# Supplementary material for: Triazine-ring protonation enables synergistic enhancement of proton conduction and membrane stability
Source: Chem Sci. 2025 Oct 31;16(47):22291–8. doi: 10.1039/d5sc05445a (PMC12577585; doi:10.1039/d5sc05445a)
Supplement: SC-016-D5SC05445A-s001 [file SC-016-D5SC05445A-s001.pdf]

Supplementary Information (SI) for Chemical Science.
This journal is © The Royal Society of Chemistry 2025

Supporting Information

Triazine-ring protonation enables synergistic enhancement of proton conduction and membrane stability

Yunfa Dong[a,†], Haodong Xie[a,†], Yupei Han[b], Quan Li[c], Jiecai Han[a], Weidong He[a,d★]

[a]National Key Laboratory of Science and Technology on Advanced Composites in Special Environments, and Center for Composite Materials and Structures, Harbin Institute of Technology, Harbin 150080, China

[b]Department of Chemistry, University College London, London WC1H 0AJ, United Kingdom

[c]Chongqing Academy of Science and Technology, Chongqing 401100, China

[d]Chongqing Research Institute, Harbin Institute of Technology, Chongqing 401151, China

Corresponding Author

★E-mail: *weidong.he@hit.edu.cn*

†These authors have contributed equally.

Experimental section

Materials

All chemicals and solvents were of reagent quality and used without further purification. Perfluorosulfonic acid (PFSA) resin was purchased from Kedi Chemical Technology Co., LTD (China). Melamine and thiocyanate were purchased from Aladdin Biochemical Technology Co., Ltd. N-Methylpyrrolidone (NMP) (>99.9%) was purchased from BASF Co. Ltd., Mainland, China. Gore-select was purchased from Alfa Aesar.

Synthesis of MT

Add equal molar amounts of melamine and thiocyanate to the flask. Keeping the solid-liquid mass ratio at 1:18, add a certain amount of deionized water, stir and react at 90 °C for 4 hours, then pump filter, wash with deionized water and dry at 80 °C for 24 hours to obtain a pale yellow powder.

Fabrication of the different PEMs

Ultrasonically disperse 0.2 g MT in 133 mL NMP to obtain a dispersion. Add 20 g perfluorosulphonic acid resin powder to the dispersion and stir magnetically until a uniform emulsion is formed, then spread the emulsion on a glass plate. First, blast dry at 80 °C for 10 hours, then vacuum dry at 120 °C for 4 hours, wash with 3 wt.% hydrogen peroxide at 80 °C for 1 hour, rinse with deionized water, then soak in 1M sulfuric acid at 80 °C for 1 hour and rinse with ionized water three times to obtain a composite proton exchange membrane with a thickness of 12-15 μm.

Characterization

The surface morphologies of all samples were investigated using scanning electron microscope (SEM, Nova NanoSEM 450 Fei, USA). Fourier-transform infrared spectroscopy (FTIR) was tested with Nicolet iS10 (Thermo Fisher Scientific, USA). An XRD spectrometer (Bruker D8, Germany) was employed for X-ray diffraction (XRD) analysis with Cu (Kα=1.54178Å) radiation source operated at 40 mA and 40 kV over the 2θ range of 10°-80°. Liduid NMR is tested by Bruker 400 MHz, of these, MT uses deuterated acetone as a solvent, and PFSA, and PFSA-MT-1% use deuterated dimethylsulfoxide as a solvent.

Electrochemical Characterization

The membrane electrode assemble (MEA) was prepared by a spray gun. The anode and cathode catalyst layers (CLs) were sprayed onto both sides of the blending membranes. Pt (Hispec10000, Johnson Matthey) loadings of the CLs on both the anode and cathode sides were 0.1 and 0.4 $mg \cdot cm^{-2}$, and the active areas of the CLs were 4 cm^2. The fabrication of the MEAs was completed through the attachment of gas diffusion layers onto both sides of the membranes. The anode was fed with H_2 (RH100%) at a flow rate of 100 sccm and the cathode was fed with O_2 (RH100%) at a flow rate of 150 sccm with a back-pressure of 14.5 PSI. The cell temperature was maintained at 70 °C. The cell performance was recorded by Arbin Fuel Cell Instrument. The hydrogen crossover of the MEAs test was conducted by using the linear sweep voltammetry (LSV) through electrochemical station CHI760E. The anode was used as the reference electrode and the counter electrode, the cathode was used as the working electrode. The cell voltage was scanned dynamically at 2 $mV \cdot s^{-1}$

in the potential range of 0-0.8 V. The anode was fed with H_2 (RH100%) at a flow rate of 200 sccm and the cathode was fed with N_2 (RH100%) at a flow rate of 200 sccm. H_2 crossover was determined by the limiting current density at higher potential (0.4 V). The oxidative stability of the fabricated composite membrane was evaluated using the Fenton method. Fresh composite membranes were dried and weighed, then immersed in 50 mL of Fenton's reagent (5 wt.% H_2O_2, 5 ppm Fe^{2+}) at 80 °C. The Fenton solution was replaced every 24 hours to maintain H_2O_2 concentration. After degradation, the membranes were rinsed with deionized water, dried, and reweighed before proceeding to the subsequent degradation cycle.

Molecular dynamics simulation

MSD calculation:

Use the construction function in the Amorphous Cell module of Materials Studio to build two models (PFSA and PFSA-MT-1%). Perform geometry optimization and annealing at 120 °C on the models sequentially using the Geometry Optimization and Anneal functions in the Forcite module. Conduct Dynamics simulations on the optimized models with the Compass III force field, using a time step of 1 fs and a total simulation duration of 5000 ps. After the simulation, perform MSD analysis on the trajectory after density stabilization.

Hydrogen bond simulation:

The polymer box was constructed using the Amorphous Cell module. Geometric optimization and molecular dynamics calculations were performed with the Forcite module, ultimately yielding the trajectory file of the polymer box under an isobaric

environment. The radial distribution function (RDF) of the box was further calculated using the Radial Distribution Function module in Forcite to determine the cutoff radius for hydrogen bonds. The Calculate Hydrogen Bonds module was employed to perform frame-by-frame analysis of the trajectory file, utilizing the hydrogen bond cutoff radius to quantify the number and length of hydrogen bonds in the first coordination sphere, thereby generating the corresponding PDF curves.

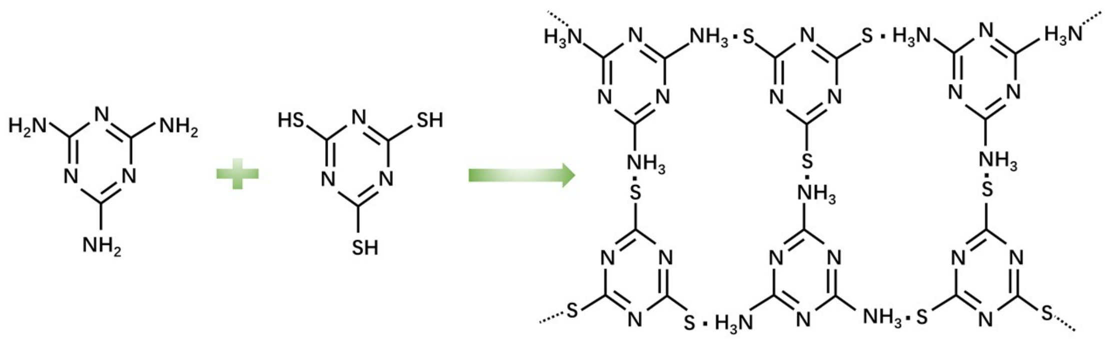

Figure S1 Molecular structural formula and synthesis reaction of MT

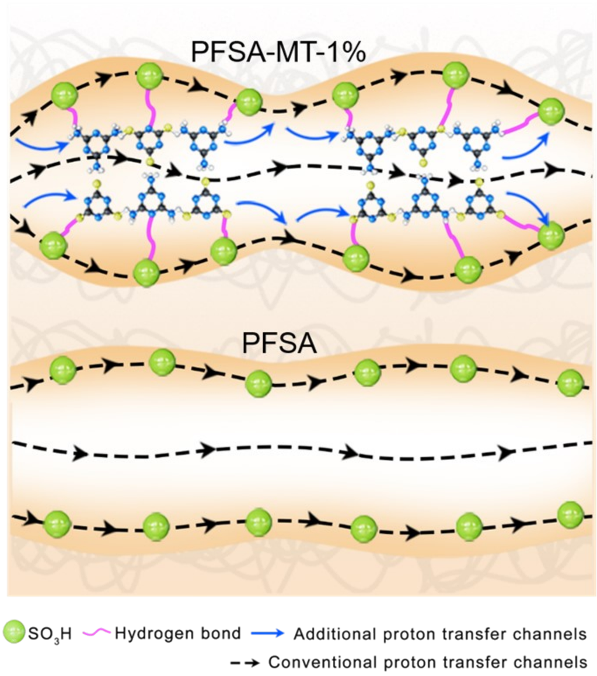

Figure S2 The proton conduction mechanism of PFSA-MT-1% and PFSA

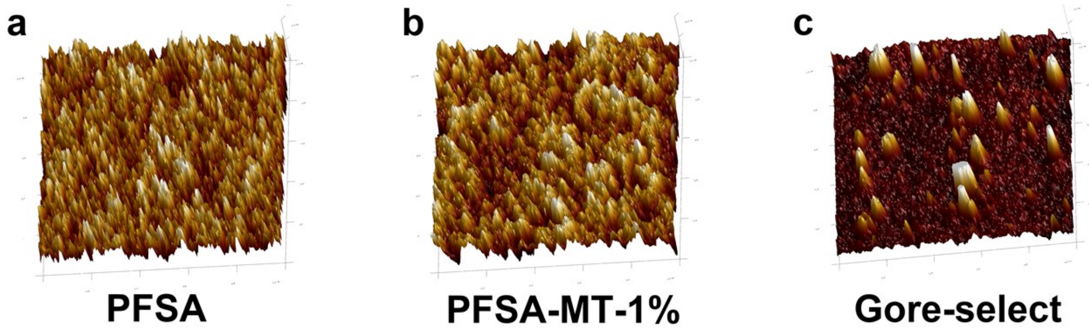

Figure S3 Roughness images of different proton exchange membranes: **(a)** PFSA, **(b)** PFSA-MT-1%, and **(c)** Gore-Select

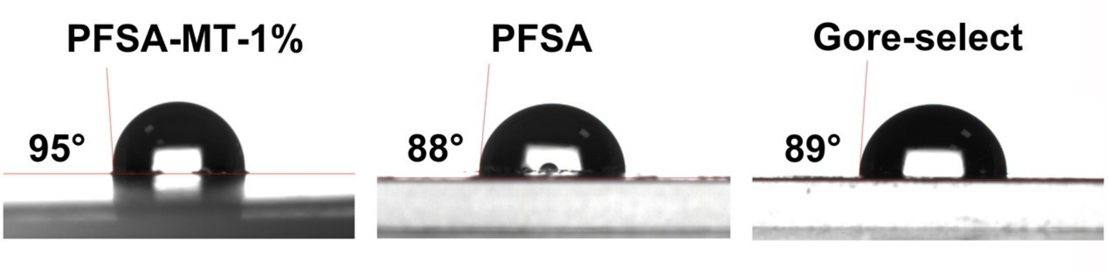

Figure S4 Photographs of contact angles of different proton exchange membranes

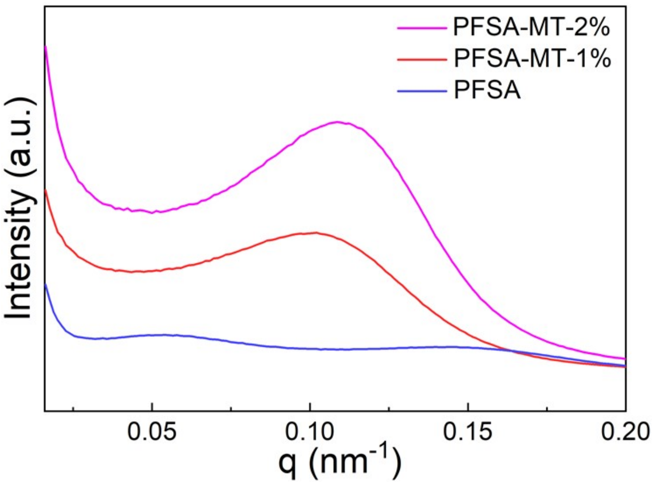

Figure S5 SAXS of different PEMs

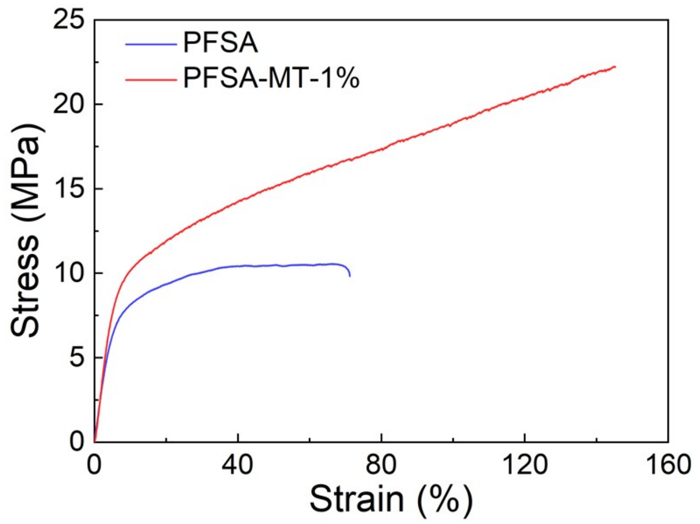

Figure S6 Strain-stress curves of PFSA and PFSA-MT-1%

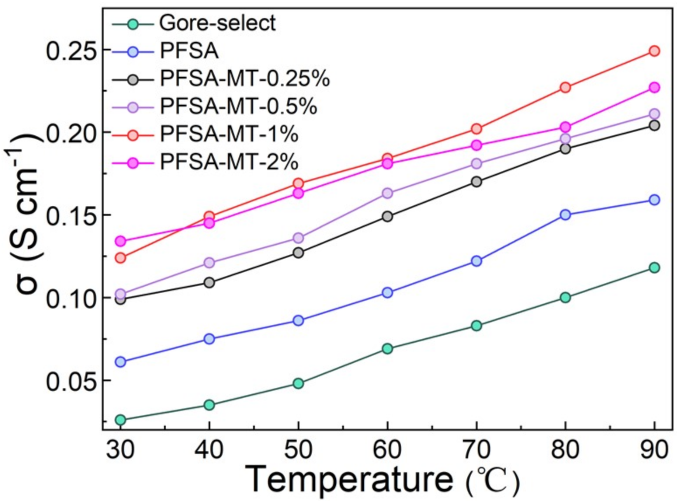

Figure S7 Proton conductivity of different membranes at different temperatures

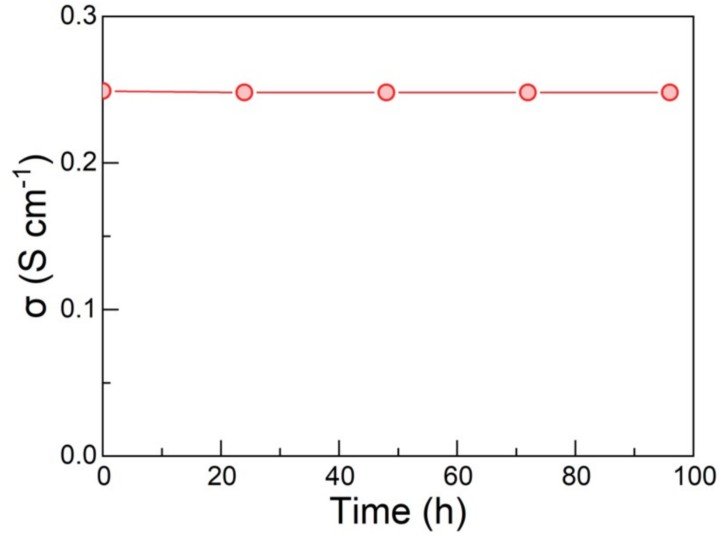

Figure S8 Time-dependent conductivity measurements at 90 °C of PFSA-MT-1%.

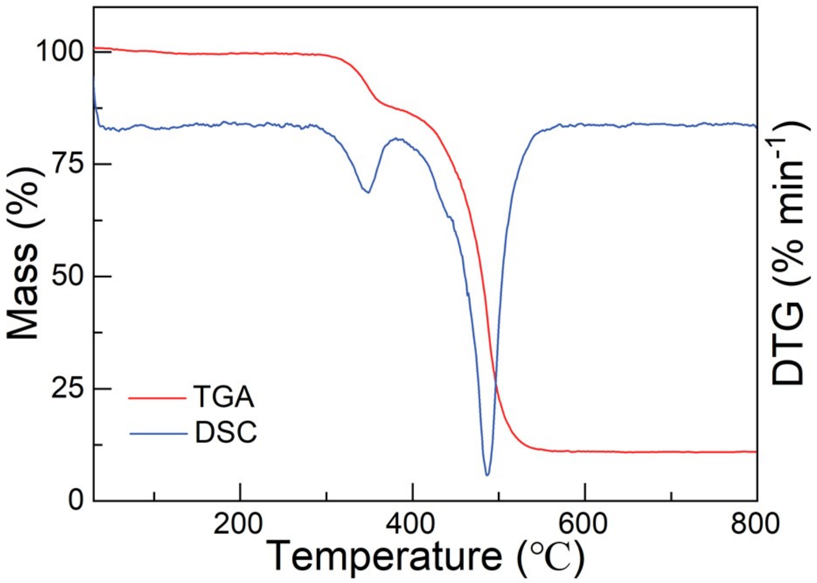

Figure S9 TGA and DSC curves of PFSA-MT-1%.
